# Multimodal fusion of liquid biopsy and CT enhances differential diagnosis of early-stage lung adenocarcinoma

**DOI:** 10.1038/s41698-024-00551-8

**Published:** 2024-02-26

**Authors:** Yanwei Zhang, Beibei Sun, Yinghong Yu, Jun Lu, Yuqing Lou, Fangfei Qian, Tianxiang Chen, Li Zhang, Jiancheng Yang, Hua Zhong, Ligang Wu, Baohui Han

**Affiliations:** 1 Department of Pulmonary Medicine, Shanghai Chest Hospital, Shanghai Jiao Tong University School of Medicine, Shanghai, China; 2 Institute for Thoracic Oncology, Shanghai Chest Hospital, Shanghai Jiao Tong University School of Medicine, Shanghai, China; 3 Dianei Technology, Shanghai, China; 4 Shanghai Lung Cancer Center, Shanghai Chest Hospital, Shanghai Jiao Tong University School of Medicine, Shanghai, China; 5 Computer Vision Laboratory, Swiss Federal Institute of Technology Lausanne (EPFL), Lausanne, Switzerland; 6 State Key Laboratory of Molecular Biology, Shanghai Key Laboratory of Molecular Andrology, Center for Excellence in Molecular Cell Science, Shanghai Institute of Biochemistry and Cell Biology, Chinese Academy of Sciences, University of Chinese Academy of Sciences, Shanghai, China

**Keywords:** Surgical oncology, Diagnostic markers, Cancer screening, Non-small-cell lung cancer

## Abstract

This research explores the potential of multimodal fusion for the differential diagnosis of early-stage lung adenocarcinoma (LUAD) (tumor sizes < 2 cm). It combines liquid biopsy biomarkers, specifically extracellular vesicle long RNA (evlRNA) and the computed tomography (CT) attributes. The fusion model achieves an impressive area under receiver operating characteristic curve (AUC) of 91.9% for the four-classification of adenocarcinoma, along with a benign-malignant AUC of 94.8% (sensitivity: 89.1%, specificity: 94.3%). These outcomes outperform the diagnostic capabilities of the single-modal models and human experts. A comprehensive SHapley Additive exPlanations (SHAP) is provided to offer deep insights into model predictions. Our findings reveal the complementary interplay between evlRNA and image-based characteristics, underscoring the significance of integrating diverse modalities in diagnosing early-stage LUAD.

Lung cancer stands as the leading cause of cancer-related deaths worldwide^1^. Early detection through low-dose CT (LDCT) has shown significant potential in reducing mortality rates, as demonstrated by notable trials such as the National Lung Screening Trial (NLST)^2^ and the Dutch-Belgian Lung Cancer Screening Trial (NELSON)^3^. One significant challenge in LDCT screening is the high rate of false-positive results, leading to unnecessary biopsy or surgical procedures. For instance, the NLST reported a false-positive rate of 26.3% for baseline screening^2^, while the NELSON trial reported a rate of 19.8%^3^. Lung nodules identified during LDCT screening, often smaller than 2 cm^4,5^, are challenging to biopsy effectively^6,7^. Therefore, the primary approach involves close monitoring, but larger tumors may exhibit resistance or metastasize during this period.

In addition to LDCT screening, liquid biopsies can identify various biomolecular features, providing potential insights into disease status^8^. Combining liquid biopsy with AI methods holds significant promise for early-stage diagnosis^9,10^. Extracellular vesicle long RNA (evlRNA), identified as a candidate biomarker, is enriched in the blood of lung cancer patients compared to healthy controls, showing significant diagnostic value in early-stage LUAD patients^11,12^. However, many current liquid biopsies focusing on early cancer detection lack the sensitivity needed for reliable identification of early-stage cancers^13^.

Artificial intelligence (AI) and biomarkers, both non-invasive, hold substantial promise in shaping the future of lung cancer screening^14^. The combined assessment of CT and evlRNA features in lung cancer cases has not been thoroughly investigated. Our study aims to explore the complementarity of these two modalities, leveraging their respective strengths and addressing individual weaknesses for the early-stage diagnosis of LUAD with tumors smaller than 2 cm.

This study enrolled 146 participants (Table S1) who underwent lung surgeries due to the presence of pulmonary nodules. These individuals had available preoperative blood samples and chest CT scans. Among them, 111 patients were diagnosed with LUAD, while 35 were categorized as benign. The LUAD group is subdivided into three pathological categories: adenocarcinoma in situ (AIS; *N* = 36), minimally invasive adenocarcinoma (MIA; *N* = 34), and invasive adenocarcinoma (IA; *N* = 41).

Model development details are illustrated in Fig. 1A. We extracted imaging features, referred to as Rad features, from a pre-trained multitask 3D DenseSharp neural network^15^. These features included malignancy probability, IA probability, invasiveness category, attenuation category, 2D diameter, and volumetric consolidation tumor ratio (vCTR). In addition, blood samples were collected in 10 mL K2EDTA anticoagulant vacutainer tubes. Subsequent steps for serum extracellular vesicle (EV) purification, RNA isolation and RNA-seq analysis followed procedures from our prior study^12^. We selected 17 evlRNA features from differentially expressed genes (DEGs) between the LUAD and control groups. Moreover, to evaluate our methods compared to human performance and investigate the potential enhancement of diagnostics through the integration of human expertise, we conducted an observer study involving both a senior and a junior investigator.

**Fig. 1:**
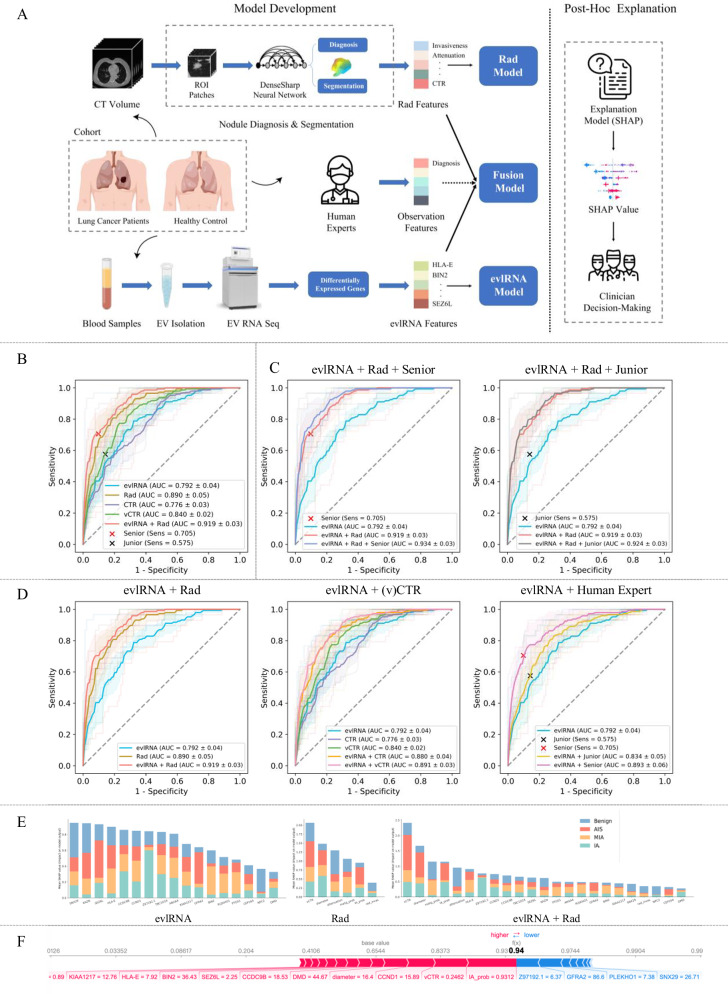
Development, diagnostic performance and post hoc explanation of the multimodal fusion model. **A** Multimodal fusion model development and post hoc explanation. **B**–**D** The validation performance evaluation of different models was conducted using 5-fold cross-validation for four-category classification (Benign AIS, MIA, IA). The mean ROC curves are depicted with dark lines, while the ROC curves for each fold are shown with light-colored lines. The shaded area surrounding the average curves indicates the standard deviation of the 5-fold. The legend for the ROC curve includes the mean AUC with median (±SD). The senior or junior models are represented by the marker “x” with their corresponding sensitivity provided in the legend. **B** Single-modal models compared with evlRNA + Rad multimodal models. **C** Collaboration of evlRNA, Rad, and human analysis from junior and senior expert. **D** Multimodal fusion of evlRNA and image-based features (Rad, (v)CTR, human expert). **E** Features importance of 4-category classification in SHAP post hoc explanation for three models (evlRNA, Rad, evlRNA + Rad). In each subplot, the horizontal axis denotes the feature names, and the vertical axis denotes SHAP values. Features with larger SHAP values are more important. The four distinct colors on the graph correspond to the four categories. Some features in the figure are abbreviated: malignancy probability (malig_prob), IA probability (IA_prob), and invasiveness classification (Rad_invas). **F** Explanation of SHAP values for a patient prediction from the model evlRNA + Rad. This patient is pathologically diagnosed as IA. The function f(x) is the output of the model (the predicted probability 0.94), and the base value follows the average of the model predictions. Features that increase the prediction (i.e., higher risk) are highlighted in red, while features that decrease the prediction (i.e., lower risk) are highlighted in blue. The size of the arrow denotes the effect of the features.

For multimodal fusion, incorporating Rad features extracted by AI from CT, evlRNA features from liquid biopsy, and observation features from clinicians, we employed the XGBoost machine learning framework^16^. Separate XGBoost models were established for each feature fusion scenario, with a primary training objective of multi-class classification (IA, MIA, AIS, Benign). The flexibility to use different combinations allows for diverse subgroup analyses. A 5-fold cross-validation approach was adopted, and average results are reported.

The performance evaluation of the multimodal fusion is shown in Fig. 1B–D, revealing several intriguing discoveries:

1. *Combining evlRNA and Rad features results in a highly effective diagnostic method*, with an impressive AUC of 0.919 (Fig. 1B). This combined model surpasses unimodal models and is comparable to the performance of senior expert.
2. *Integrating human expertise with the combination of evlRNA and Rad characteristics leads to improved results*, with AUC values of 0.934 and 0.924 for the inclusion of senior and junior experts, respectively (Fig. 1C).
3. *Furthermore, evlRNA-based and image-based features complement each other, displaying a mutually reinforcing relationship* (Fig. 1D). The three subplots illustrate that combining evlRNA with image-based attributes (Rad, (v)CTR, observer) leads to better performance than using a single modality.

The evlRNA + Rad model outperforms other multi-modal fusion models without human expert intervention. In the subsequent text, we’ll use it as our standard fusion model. We conducted a detailed assessment of our model’s performance, concentrating on three vital clinical subtasks (see Table 1):

- The binary classification distinguishing between malignant nodules (IA, MIA, or AIS) and benign nodules. The fusion model attained an impressive area under receiver operating characteristic curve (AUC) of 94.8%, with sensitivity of 89.1% and specificity of 94.3%.
- The binary classification distinguishing between invasive nodules (IA or MIA) and preinvasive nodules (AIS or Benign). The goal is to reduce overdiagnosis in line with the 2021 WHO guidelines^17^. Invasive nodules require surgical intervention due to their worse prognosis, while preinvasive nodules usually need CT monitoring. The fusion model performed well in this task with an AUC of 87.2%, a sensitivity of 80.0%, and a specificity of 87.1%.
- The binary classification distinguishing IA nodules from MIA in invasive nodules. MIA patients have a high disease-free survival rate, nearly 100%, with careful limited resection^18^. In contrast, IA patients have a lower disease-free survival rate, around 60% to 70%^19,20^. The fusion model achieved excellent results for this task with an AUC of 92.1%, a sensitivity of 92.8%, and a specificity of 88.6%.

**Table 1:** Classification performance overall and across three clinical subtasks

Method	Category Classification	Subtasks analysis									
		(IA, MIA, AIS) vs Benign				(IA, MIA) vs (AIS, Benign)			IA vs MIA		
	AUC	*P*	AUC	Sens	Spec	AUC	Sens	Spec	AUC	Sens	Spec
*Single-modal analysis*											
evlRNA	79.2 (75.0– 83.4)	0.001	86.4 (82.3 –90.4)	83.9 (78.2– 89.5)	85.7 (85.7– 85.7)	75.8 (64.4– 87.3)	73.3 (48.2– 98.5)	78.9 (68.3– 89.6)	81.7 (76.4– 86.9)	75.8 (59.0– 92.7)	91.4 (80.2– 102.6)
CTR	77.6 (74.4– 80.8)	0.001	75.7 (71.6– 79.8)	59.5 (50.5– 68.5)	94.3 (87.4– 101.1)	62.9 (60.1– 65.7)	77.3 (59.5– 95.1)	59.2 (47.1– 71.2)	73.7 (57.8– 89.6)	82.5 (67.8– 97.2)	72.4 (48.8– 95.9)
vCTR	84.0 (82.0– 86.0)	<0.001	85.1 (81.6– 88.5)	71.0 (57.2– 84.9)	97.1 (91.5– 102.7)	75.9 (69.8– 82.0)	68.0 (51.3– 84.7)	80.3 (68.2– 92.3)	79.3 (75.9– 82.7)	70.6 (57.8– 83.3)	93.8 (86.3– 101.3)
Rad	89.0 (84.4– 93.6)	<0.001	92.2 (87.0– 97.5)	79.2 (69.5–88.8)	94.3 (87.4–101.1)	82.9 (74.6–91.2)	76.0 (63.2–88.8)	85.8 (75.8–95.8)	89.2 (81.6–96.9)	87.8 (76.8–98.8)	94.3 (87.4–101.1)
Junior expert	/	/	/	83.7 (76.4– 91.0)	51.4 (30.5– 72.4)	/	76.0 (66.2– 85.8)	66.2 (58.0– 74.3)	/	81.1 (64.5– 97.7)	79.5 (65.4– 93.6)
Senior expert	/	/	/	91.9 (87.5– 96.3)	80.0 (73.1– 86.8)	/	85.3 (75.7– 94.9)	77.4 (74.4– 80.4)	/	85.6 (76.9– 94.2)	76.7 (57.9– 95.4)
*EvlRNA-based and image-based features are complementary*											
evlRNA + CTR	88.0 (84.5– 91.5)	<0.001	90.5 (87.1– 93.9)	87.4 (78.0– 96.9)	88.6 (83.0– 94.2)	84.0 (77.5– 90.4)	72.0 (60.8– 83.2)	90.0 (81.6– 98.4)	88.1 (83.7– 92.6)	90.3 (81.2– 99.4)	82.4 (72.0– 92.8)
evlRNA + vCTR	89.1 (86.2– 92.0)	<0.001	91.0 (86.8– 95.2)	84.6 (77.3– 91.9)	91.4 (84.6– 98.3)	82.7 (74.1– 91.2)	68.0 (49.8– 86.2)	91.4 (80.2– 102.6)	90.7 (85.6– 95.8)	92.8 (87.0– 98.6)	85.2 (76.3– 94.1)
**evlRNA + Rad**	91.9 (88.6– 95.2)	<0.001	94.8 (91.3– 98.3)	89.1 (83.1– 95.1)	94.3 (87.4– 101.1)	87.2 (80.2– 94.3)	80.0 (63.0– 97.0)	87.1 (70.3– 104.0)	92.1 (88.5– 95.7)	92.8 (87.0– 98.6)	88.6 (78.1– 99.0)
evlRNA + Junior expert	83.4 (78.7– 88.1)	<0.001	89.9 (86.3– 93.5)	86.4 (78.9– 93.9)	88.6 (78.1– 99.0)	82.8 (77.8– 87.8)	73.3 (57.9– 88.8)	84.5 (73.2– 95.7)	82.1 (73.1– 91.1)	88.1 (77.7– 98.4)	77.1 (58.2– 96.1)
evlRNA + Senior expert	89.3 (83.9– 94.7)	<0.001	95.1 (91.1– 99.2)	86.5 (78.0– 95.0)	97.1 (91.5– 102.7)	87.8 (81.0– 94.6)	86.7 (78.4– 95.0)	80.2 (68.9– 91.5)	84.2 (76.0– 92.4)	75.8 (63.1– 88.6)	85.7 (64.0– 107.4)
*Multimodal + Human analysis collaboration further improves performance*											
evlRNA + Rad + Junior expert	92.4 (89.2– 95.6)	<0.001	96.0 (92.7– 99.2)	95.5 (91.5– 99.4)	91.4 (84.6– 98.3)	87.3 (79.2– 95.5)	78.7 (63.5– 93.8)	88.6 (79.1– 98.1)	92.5 (90.2– 94.8)	90.3 (81.2– 99.4)	91.4 (84.6– 98.3)
evlRNA + Rad + Senior expert	93.4 (90.5– 96.3)	<0.001	97.9 (96.0– 99.9)	91.8 (85.3– 98.4)	100.0 (100.0– 100.0)	88.9 (81.6– 96.1)	92.0 (84.4– 99.6)	80.1 (65.0– 95.1)	92.4 (89.0– 95.9)	88.1 (77.7– 98.4)	94.3 (87.4– 101.1)

including discrimination between malignant nodules (IA, MIA, or AIS) and benign nodules, discrimination between invasive nodules (IA or MIA) and preinvasive nodules (AIS or Benign), and discrimination between IA nodules and MIA nodules. The performance metrics of AUC (%), AUC *p*-value (P), sensitivity (Sens, %), and specificity (Spec, %) are presented from the mean of 5-fold cross-validation, with 95% confidence interval (CI) provided. Bold: Best-performing model within its subtasks and subgroup analysis.

Our fusion model consistently outperforms single-modal models across different subtasks, just as it did in the four-class classification. Notably, our fusion method significantly improves specificity, effectively reducing false positives and overdiagnosis. The fusion model exceeds the specificity of senior experts by 14.3%, 9.7%, and 11.9% in subtasks (a), (b), and (c), respectively. Furthermore, when combining evlRNA, Rad, and senior expert inputs, our model achieves 100% specificity in distinguishing malignant from benign nodules during cross-validation.

To enhance the understanding of feature importance in predictive modeling, we employed the SHapley Additive exPlanations (SHAP) post hoc explanatory framework^21^. We applied this framework to three models: evlRNA, Rad, and evlRNA + Rad. The feature impacts for the 4-category classification are depicted in Fig. 1E. Notably, in the fusion model, vCTR is the most crucial feature. Furthermore, the SHAP framework extended to individualized validation predictions (Fig. 1F). The visual illustration unveils that the patient with a high probability of 0.94 for being classified as IA. This probability primarily results from factors such as an IA probability of 0.9312, a vCTR value of 0.2426, a gene CCND value of 15.89, and other risk-contributing factors. Understanding individual predictions is valuable for clinical decision-making. In addition, we explored how feature values relate to predicted categories (Fig. S1 in Supplementary). In the evlRNA analysis, certain genes exhibit distinct correlations with category predictions, which become more evident in the Rad feature analysis. We believe Rad features, being AI-generated, naturally possess discriminative abilities. In the joint analysis of Rad and evlRNA features, the top five crucial features combine genetic and imaging traits, highlighting their synergistic effects (details in Supplementary Results).

Assessing a model’s robustness is crucial for both evaluation and practical use. We evaluated the robustness of our XGBoost model by adding Gaussian noise to input features (Fig. S2). With low noise, the model’s performance slightly declines, but as noise increases, the degradation intensifies. Remarkably, in a specific noise range, our multimodal fusion model, consistently outperforms single-modal models, showcasing its robustness.

Our study has a few limitations. Firstly, we only include 146 participants due to difficulties in obtaining both evlRNA detection data and CT imaging samples. Collecting evlRNA information is time-consuming and expensive. In the future, a larger dataset is needed to avoid overfitting and improve validation accuracy. Secondly, our study only involved internal validation and did not include external validation, thereby leaving the model’s applicability and generalizability unexplored.

In summary, our study has underscored the complementary nature between evlRNA-based and image-based features, with human analysis integration leading to improved performance. These results emphasize the critical importance of multimodal fusion to enhance differential diagnosis of early-stage lung adenocarcinoma in the LDCT screening.

## Methods

### Data characteristics

The study involved 146 participants who underwent lung operations due to the presence of pulmonary nodules between 2018 and 2020. This group included 111 patients diagnosed with lung adenocarcinoma (LUAD) and 35 controls classified as benign cases. Essential participant characteristics are provided in Table S1. The following inclusion criteria for the LUAD patients were applied: (a) patients pathologically proven to have LUAD (tumor size < 2 cm), (b) obtainable preoperative blood samples, (c) obtainable chest CT scan, and (d) patients gave their informed consent before enrollment.

This study was approved by the ethics committee of Shanghai Chest Hospital, Shanghai Jiao Tong University School of Medicine, and complied with all relevant ethical regulations including the Declaration of Helsinki. All participants were from a registered lung cancer screening study (China Lung Cancer Screening Study, NCT03975504), and signed informed consent to take part in the research. Our study did not specifically address cases involving multiple nodules. In our cohort, only two individuals had multiple pulmonary nodules. For these cases, we chose to analyze only the most severe nodule.

In this study, the CT images of the latest CT examination before surgery were collected from a single clinical center (Chest Hospital affiliated to Shanghai Jiaotong University School of Medicine). Thicknesses of these scans range between 0.625 mm and 1.5 mm. The pathological label and mass center of each lesion is manually labeled by a junior thoracic radiologist, according to corresponding pathological reports. These annotations are then confirmed by a senior radiologist with 15 years of experience in chest CT. Patient identities are anonymized for privacy protection.

### Pre-trained DenseSharp model

To extract nodule features from CT images, we utilized a pre-trained 3D DenseSharp neural network^15^, which had undergone extensive training on two internal datasets: Pretraining cohort A contained 651 subcentimeter nodules^15^, and pretraining cohort B comprised 4728 nodules from the Pulmonary-RadPath dataset^22^. Number of nodules for pretraining can be found in Table S2. The DenseSharp model generates outputs through five heads: four for classification and one for creating a 3D nodule segmentation. The four classification tasks include invasiveness with four categories (Benign, AIS, MIA, IA), malignancy (benign/malignant), IA (non-IA/IA), and attenuation with three categories (solid, part-solid, ground-glass).

We conducted standard data preprocessing adhering to common practices: (1) Resampling CT volumes to dimensions of 1 mm × 1 mm × 1 mm. (2) Normalizing Hounsfield Units to the range [−1, 1]. (3) Cropping a 32 × 32 × 32 volume centered at the centroid of each lesion. In our proposed model, the input consists of a cubic CT volume patch measuring 32 mm × 32 mm × 32 mm.

The training employs early stopping based on validation loss—training stops if the validation loss does not decrease within 10 epochs. We incorporate online data augmentations, such as random rotation, flipping, and translation, in every volume. We use Adam optimizer^23^ to train all models end-to-end for 200 epochs. Our experiments are conducted using PyTorch 1.11^24^ on 2 Nvidia RTX 3090 GPUs.

### Extracting imaging features

We employed the pre-trained 3D DenseSharp neural network to perform the classification task and generate the nodule mask. We collected prediction logits from the classification task, resulting in four nodule attributes. Since the size of the solid component within SSNs observed on CT images is closely related to the extent of tumor infiltration^25,26^, we developed an internal tool to calculate 2D diameter (mm), the consolidation tumor ratio (CTR), and volumetric CTR (vCTR). Notably, a nuanced differentiation exists between the two, as CTR measures the diameter fraction of the solid components in nodules, whereas vCTR quantifies the volumetric proportion. By combining these features with the previously established fundamental attributes, we derived a set of six nodule imaging features known as Rad features. These Rad features encompass malignancy probability, IA probability, invasiveness category, attenuation category, clinically measured 2D diameter (mm), and v(CTR).

### Extracting evlRNA features

The methodologies employed for collection of blood samples, serum extracellular vesicle (EV) purification, RNA isolation, characterization of EVs, construction of evlRNA libraries and subsequent RNA-seq analysis closely adhere to those detailed in the previously cited work^12^. We explored the differentially expressed genes (DEGs) between the LUAD and control groups, which revealed a total of 145 upregulated and 363 downregulated DEGs (*p* value < 0.05, fold change > 1.5). Feature selection was performed by the Boruta algorithm^27^ to find all relevant variables for machine learning. A signature of 17 DEGs were selected as diagnostically informative EV-associated evlRNAs: HLA-E, BIN2, Z97192.1, KAZN, CCDC9B, PLEKHO1, PTGS1, ANXA4, SNX29, CEP164, GFRA2, TBC1D24, NPC2, CCND1, KIAA1217, DMD, and SEZ6L.

### Integrating multimodality features

We employed the machine learning framework XGBoost^16^ to perform multimodal fusion of various features. To integrate human expertise, we gathered pathological four-type judgments from both doctors for all samples. These judgments were then used as features, combined with other modal features, and introduced as fusion features into the XGBoost model for training and validation. In our experiments, we established separate XGBoost models for each feature fusion scenario.

The primary training objective of our model involves multi-class classification, specifically distinguishing between (IA, MIA, AIS, Benign) categories. This is achieved using the multiclass softmax as the objective function, which generates a probability distribution for each class. When it comes to prediction results, we have the flexibility to use different combinations based on specific needs, allowing for various subgroup analyses. As an illustration, we outline the calculation of positive and negative probabilities for each task as follows: in task (a), y_postive_ = y^IA^ + y^MIA^ + y^AIS^, y_negative_ = y^Benign^; in task (b), y_postive_ = y^IA^ + y^MIA^, y_negative_ = y^AIS^ + y^Benign^; in task (c), y_postive_ = y^IA^, y_negative_ = y^MIA^.

We adopted a 5-fold cross-validation approach, wherein the entire dataset was evenly divided into five distinct subsets. During each iteration, four subsets were used for training, leaving one subset for validation. The stopping criteria involve early stopping based on the maximum number of iterations, with the default value of num_boost_round set to 10. The reported performance metrics represent the average results obtained across the fivefold validation. To assess the effectiveness of a diagnostic test in distinguishing between positive and negative cases, we employed the Youden index. This threshold is employed to strike a balance between sensitivity and specificity. Our multi-modal fusion model’s parameters are shown in Table S3.

### Observation study

To compare our methodologies with human proficiency, an experienced senior radiologist (with over a decade of expertise in chest CT interpretation) and a junior radiologist (with 3 years of experience in chest CT interpretation) from Chest Hospital affiliated to Shanghai Jiaotong University School of Medicine were consulted. These professionals, who were kept unaware of the histopathological findings and clinical information, independently undertook the task of classifying and diagnosing all the nodules. The outcomes of their expert-based image interpretations were referred to as observation features.

### Model robustness analysis

During the experiments, we introduced Gaussian noise with a mean of 0 and observed its impact on the model’s performance (4-categoray classification AUC) as perturbations increased, corresponding to an increase in the standard deviation. We selected three models for analysis: Rad, evlRNA, and a multi-modal fusion model, evlRNA + Rad. In our study, we injected Gaussian noise into the input features of the XGBoost model with a mean of 0 and a standard deviation of σ. We then observed the trend of 5-fold AUC validation performance on the dataset as the standard deviation varied. For each standard deviation value selected, we randomly generated Gaussian noise 100 times and calculated the average AUC results for these 100 runs. In Fig. S3, we represented the average results of the 100 runs with a solid line, and we used shading to indicate the standard deviation interval for 100 noise injections.

### Reporting summary

Further information on research design is available in the Nature Research Reporting Summary linked to this article.

### Supplementary information

Reporting Summary

Supplemental materials

## Data Availability

The sequencing data have been deposited in the National Center for Biotechnology Information Gene Expression Omnibus (GEO) (http://www.ncbi.nlm.nih.gov/geo/) database under accession number GSE200288. Additional data utilized and/or analyzed during the current study are available from the corresponding author upon reasonable request.

## Code availability

Our pipeline is available at https://github.com/yinghyu5214/fusion.
