# Supplementary material for: Multimodal fusion of liquid biopsy and CT enhances differential diagnosis of early-stage lung adenocarcinoma
Source: NPJ Precis Oncol. 2024 Feb 26;8:50. doi: 10.1038/s41698-024-00551-8 (PMC10897137; doi:10.1038/s41698-024-00551-8)
Supplement: Supplementary file 2 — Supplemental materials [file 41698_2024_551_MOESM2_ESM.pdf]

Supplementary Materials:

1. Supplementary Results
2. Table S1
3. Table S2
4. Table S3
5. Figure S1
6. Figure S2

Supplementary Results

Explanation of Results in Fig. S1.

(1) In the prediction interpretation of evlRNA in Fig. S1 (A), we can observe a clear positive or negative correlation between most genes and the predicted categories. This indicates that the selected genes can effectively assist in determining the model's classification. Specifically, the genes HLA-E and BIN2 exhibit a positive correlation with the predicted results in the Benign and AIS categories, while they display a negative correlation in the MIA and IA categories. This suggests a potential negative association between these genes and the degree of invasiveness. On the other hand, the genes KIAA1217, CCND1 and Z97192.1 demonstrate a negative correlation with the predicted results in the Benign and AIS categories, but a positive correlation in the MIA and IA categories. This implies a potential positive relationship between these genes and the degree of invasiveness.

(2) In the prediction interpretation of Rad in Fig. S1 (B), features exhibit distinct positive or negative correlations with category predictions. We hypothesize that

Rad features are generated by AI and that the features themselves inherently hold discriminatory capabilities. The assessment of malignancy probability and IA probability aligns with expectations: in classifications leaning towards benign cases, there is a negative correlation with the predicted results, while in classifications leaning towards malignant cases, there is a positive correlation with the predicted results. Furthermore, vCTR and diameter features demonstrate a positive correlation with the predicted results in the Benign and IA categories, and a negative correlation in the AIS and MIA categories. However, not all samples conform strictly to positive or negative correlation distributions, possibly due to the limited number of samples. Additionally, in practical clinical settings, the proportion of solid components and the size of the diameter do not directly predict the benign or malignant nature of nodules.

The proportion of solid components is indeed related to the malignancy of nodules, as malignant nodules typically have a higher proportion of solid components. However, a high proportion of solid components alone cannot determine the benign or malignant nature of a nodule, as some benign lesions can also increase the proportion of solid components. Similarly, the diameter of a nodule is also related to its benign or malignant nature, as malignant nodules tend to be larger than benign nodules due to the higher proliferative capacity and invasiveness of cancer cells. However, some benign nodules can also be large, while early-stage malignant nodules can be small.

Relying solely on the proportion of solid components and the diameter is

insufficient to determine the benign or malignant nature of a nodule. Therefore, when evaluating the malignancy of nodules, doctors usually consider multiple factors, including the patient's medical history, clinical presentation, size of the nodule, morphological features, growth rate, clarity of the borders, and characteristics of the solid component. Additionally, further examinations such as imaging studies and possible biopsies may be required to further assess the benign or malignant nature of a nodule.

(3) In the prediction interpretation of evlRNA + Rad in Fig. S1 (C), we observe that the top five important features include both gene and imaging features. This indicates that both imaging and gene features play crucial roles in the model and exhibit complementary effects. These results suggest that gene features and imaging features provide informative contributions to nodule classification and prediction, offering different aspects of information to assist the model in making accurate predictions. Gene features provide information about the genetic characteristics and biological functions of the nodules, while imaging features reflect the morphology, density, texture, and other characteristics of the nodules in medical imaging. By combining gene features and imaging features, the model can obtain more comprehensive and integrated information, thereby improving its ability to assess the benign or malignant nature of nodules. This complementary effect helps enhance the performance and stability of the model, resulting in improved accuracy in nodule classification and prediction. Therefore, the integration and analysis of gene features and imaging features hold

significant importance in clinical applications, providing physicians with comprehensive decision-making support and assisting in the assessment of nodule malignancy and treatment selection.

Table S1. Characteristics of lung adenocarcinoma patients and controls.

AIS: adenocarcinoma in situ, MIA: minimally invasive adenocarcinoma, IA: invasive adenocarcinoma

Characteristics	Patients (N=111)	Controls (N=35)
Age (years), mean (SD)	54.7 (11.9)	54.2 (11.0)
Sex, N (%)		
Female	88 (79.3)	18 (48.6)
Male	23 (20.7)	17 (51.4)
Smoking status, N (%)		
Ever smokers	19 (17.1)	13 (37.1)
Never smokers	92 (82.9)	22 (62.9)
Size of Nodules, N (%)		
⩾5-10 mm	50 (49.5)	15 (42.8)
10-20 mm	61 (50.5)	20 (57.2)
Density of Nodules, N (%)		
Solid	22 (19.8)	-
Part-solid	30 (27.0)	-
Ground-glass	59 (53.2)	-
Pathology		
AIS	36 (32.4)	-
MIA	34 (30.6)	-
IA	41 (37.0)	-

Table S2. Number of nodules for pretraining and multimodal training

	Pretraining Cohort A[1]	Pretraining Cohort B[2]	Pretraining Total	Multimodal Cohort
Benign	39	1064	1103	35
AIS	166	1524	1690	36
MIA	316	369	685	34
IA	130	1771	1901	41
Total	651	4728	5379	146

Table S3. Multi-modal fusion model training parameters.

Training Config	parameters
n_estimators	300
learning_rate	0.1
booster	gbtree
objective	binary:logistic
gamma	0.1
max_depth	4
reg_alpha	0
reg_lambda	2
subsample	0.6
colsample_bytree	0.5
min_child_weight	1
random_state	42

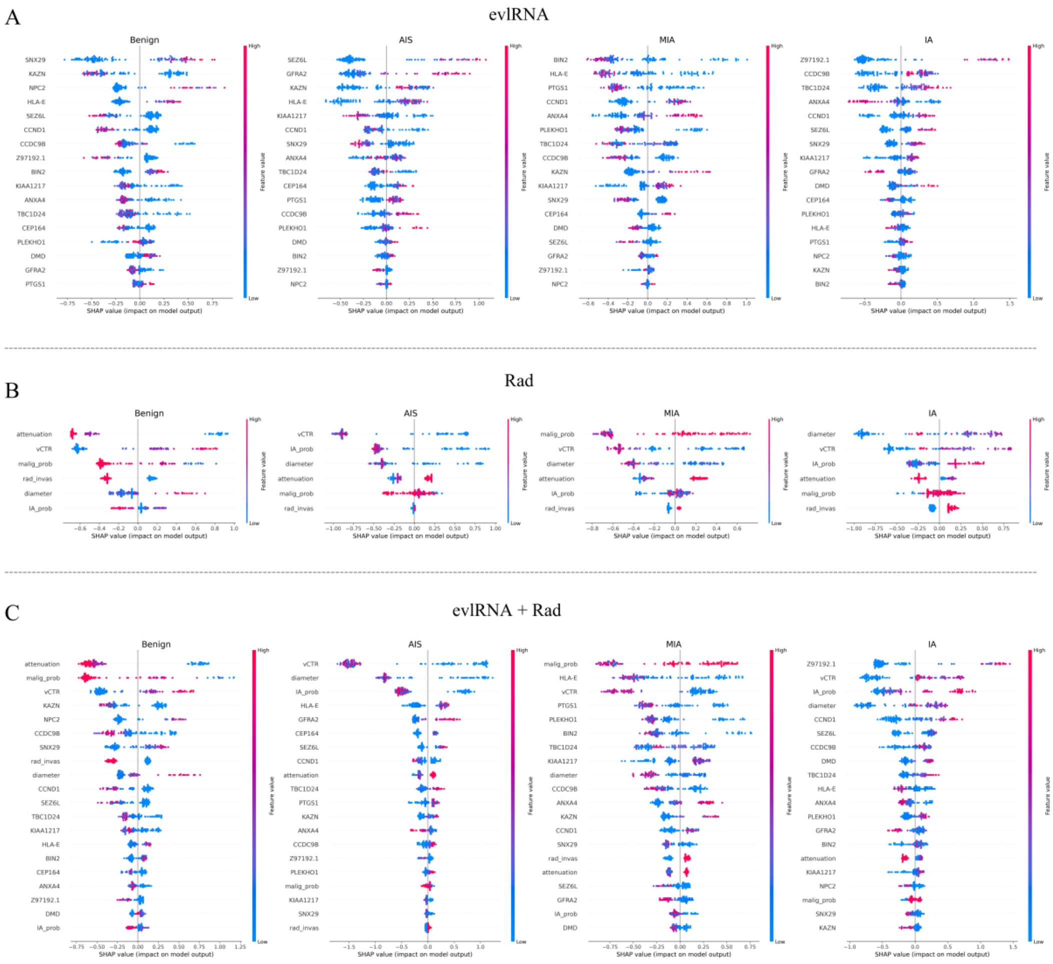

Fig. S1. Local explanation summary of 4-category classification in validation set for three models (evlRNA, Rad, evlRNA + Rad). In each subplot, the horizontal axis denotes the SHAP values, and the vertical axis denotes feature names, which is ordered according to its importance. Each data point denotes the effect of a feature on the samples. The SHAP value with less than 0 indicates a negative contribution (decrease the prediction value), equal to 0 indicates no contribution, and greater than 0 indicates a positive contribution (increase the prediction value).

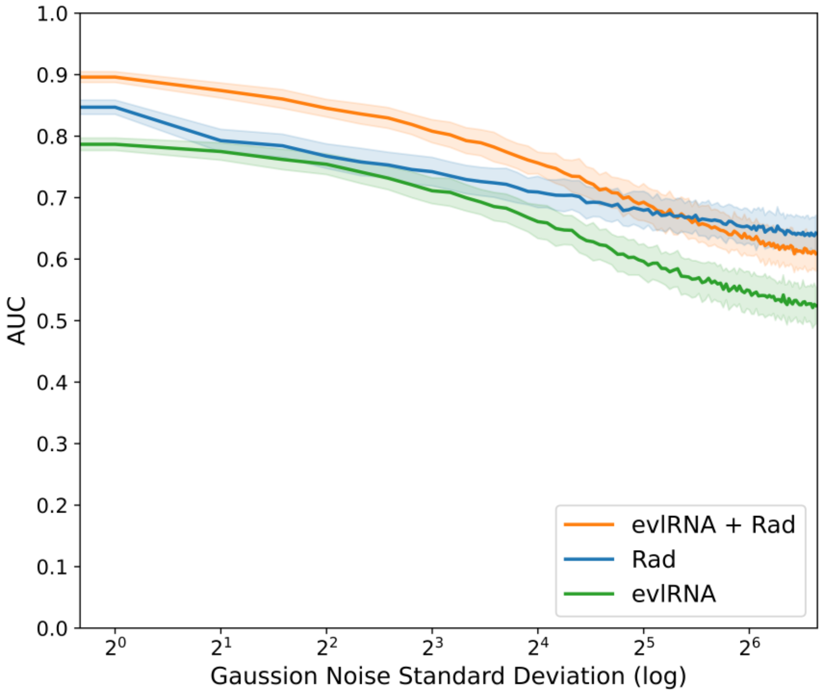

Fig. S2. Robustness analysis of evlRNA, Rad and evlRNA + Rad model. The model's classification AUC (Area Under the Curve) varies with an increase in the standard deviation of Gaussian noise added to the input features. The mean AUC curves are depicted with dark lines, while the shaded area surrounding the average curves indicates the standard deviation of the 100 random noise injections.

References

1. Zhao, W., *et al.* 3D deep learning from CT scans predicts tumor invasiveness of subcentimeter pulmonary adenocarcinomas. *Cancer research* **78**, 6881-6889 (2018).
2. Yang, J., *et al.* Hierarchical classification of pulmonary lesions: a large-scale radio-pathomics study. in *Medical Image Computing and Computer Assisted Intervention –MICCAI 2020: 23rd International Conference, Lima, Peru, October 4 –8, 2020, Proceedings, Part VI 23* 497-507 (Springer, 2020).
